# Fast Visible-Light Photopolymerization in the Presence of Multiwalled Carbon Nanotubes: Toward 3D Printing Conducting Nanocomposites

**DOI:** 10.1021/acsmacrolett.1c00758

**Published:** 2022-02-10

**Authors:** Antonela Gallastegui, Antonio Dominguez-Alfaro, Luis Lezama, Nuria Alegret, Maurizio Prato, María L. Gómez, David Mecerreyes

**Affiliations:** † POLYMAT, University of the Basque Country UPV/EHU, Avenida Tolosa 72, 20018 Donostia-San Sebastian, Gipuzkoa, Spain; ‡ Center for Cooperative Research in Biomaterials (CIC biomaGUNE), Basque Research and Technology Alliance (BRTA), 20014 Donostia-San Sebastián, Spain; § Departamento de Química Inorgánica, Facultad de Ciencias, UPV/EHU, Aptdo. 644, 48015 Bilbao, Spain; ∥ Department of Chemical and Pharmaceutical Sciences, INSTM Unit of Trieste, University of Trieste, Via L. Giorgieri 1, 34127 Trieste, Italy; ⊥ Instituto de Investigaciones en Tecnologías Energéticas y Materiales Avanzados (IITEMA) and Consejo Nacional de Investigaciones Científicas y Tecnológicas (CONICET), Campus Universitario, 5800 Universidad Nacional de Rio Cuarto, X5804 Rio Cuarto, Argentina; # IKERBASQUE, Basque Foundation for Science, 48009 Bilbao, Spain

## Abstract

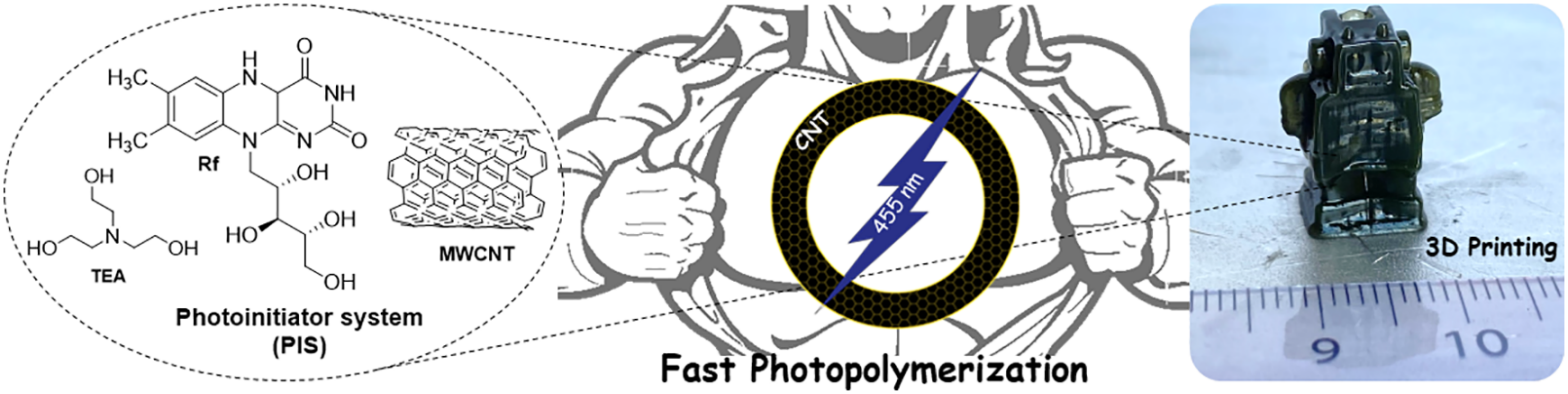

A new photoinitiator system (PIS) based on riboflavin (Rf), triethanolamine, and multiwalled carbon nanobutes (MWCNTs) is presented for visible-light-induced photopolymerization of acrylic monomers. Using this PIS, photopolymerization of acrylamide and other acrylic monomers was quantitative in seconds. The intervention mechanism of CNTs in the PIS was studied deeply, proposing a surface interaction of MWCNTs with Rf which favors the radical generation and the initiation step. As a result, polyacrylamide/MWCNT hydrogel nanocomposites could be obtained with varying amounts of CNTs showing excellent mechanical, thermal, and electrical properties. The presence of the MWCNTs negatively influences the swelling properties of the hydrogel but significantly improves its mechanical properties (Young modulus values) and electric conductivity. The new PIS was tested for 3D printing in a LCD 3D printer. Due to the fast polymerizations, 3D-printed objects based on the conductive polyacrylamide/CNT nanocomposites could be manufactured in minutes.

In the past years, the emergence of light-emitting diodes (LEDs) and the use of visible light have accelerated the development of photopolymerization methods.^1−3^ This irradiation system presents important benefits when compared to UV lamps or lasers: LEDs do not cause damage to the skin and eyes when used; their energy consumption is low (with respect to other irradiation sources); and they are environmentally friendly (they do not produce ozone or involve Hg), more economical, compact, and characterized by a long half-life time. The photopolymerization reactions using visible light are usually promoted by Type II photoinitiator systems (PISs), where the presence of two main actors is necessary: a sensitizer (S) responsible for absorbing visible light (400–700 nm) and a co-initiator which generates the active radicals able to initiate the polymerization.^4−6^ Type II PISs are more delicate and often require the absence of oxygen (a radical polymerization inhibitor), the absence of absorbent additives that could compete for light absorption, and a necessary prolonged light exposure time for quantitative polymerization. To avoid these drawbacks, novel PISs have been developed, such as macromolecular photoinitiators, photoactive polymers, and functionalized monomers, in order to obtain fast photopolymerizaiton processes. These PIS systems are being used to obtain multifunctional materials, new inks, and formulations for light-induced additive manufacturing 3D printing methods.^1,4,6−13^

Photopolymerization is also a popular technique to obtain nanocomposite materials thanks to the afforded temporal and spatial control of this in situ process and the facility to disperse the nanofillers in the liquid monomers before polymerization.^14^ One of the most popular nanofillers in polymer nanocomposites is carbon nanotubes (CNTs).^15^ Small amounts of CNTs, normally between 0.1 and 5 wt %, can not only significantly improve the mechanical and thermal properties but also provide electrical conductivity to the polymer.^16^ However, the photopolymerization of monomer/CNT formulation typically shows low conversions since CNTs can absorb light competing with the photoinitiators and limiting the initiation efficiency. Interestingly, it has also been observed that CNTs can participate directly in the mechanism of the photopolymerization reaction. For instance, Guo et al. proposed radical initiator generation by single-walled CNTs (SWCNTs) in the photoinitiated thiol–ene polymerization process employing visible light, reaching a polymerization conversion of up to 80%.^17^ In another example, Sangermano et al. studied the UV-photopolymerization process employing SWCNTs as photoinitiators with a final improved conversion of 60% and also investigated the incorporation of MWCNTs in visible-light photopolymerization through a cationic mechanism for the synthesis of epoxides, achieving a 75% conversion in 30 min.^18,19^ However, these photopolymerization processes are still far from the fast kinetics and quantitative conversions needed for additive manufacturing technologies such as stereolithographic (SLA), digital light processing (DLP), or liquid crystal display (LCD) 3D printing.^20−22^

The goal of this letter is to propose a new fast visible-light photopolymerization initiator system in the presence of CNTs which allows the preparation of conducting polymer nanocomposites and objects by LCD 3D printing.^23−25^ Our aim is to extend the range of conducting polymer materials for additive manufacturing.^26−28^

The visible-light photopolymerization initiation system (PIS) is composed of vitamin B2 (riboflavine, Rf) as the sensitizer, triethanolamine as a co-initiator (TEA), and MWCNTs as catalysts as illustrated in Scheme 1. This new type II PIS MWCNT/Rf/TEA was employed in the polymerization of acrylamide (AAm) with a small amount of PEGDA cross-linker in 50 wt % solid content water formulation, employing 0.25 wt % of MWCNTs with respect to the monomer. Using a homemade portable irradiator equipped with three blue LEDs (emission maximum = 455 nm), the photopolymerization proceeded almost instantaneously and quantitatively. As a result, polyacrylamide (PAAm) hydrogels containing MWCNTs were obtained. On the contrary, using the typical type II initiation system Rf/TEA without MWCNTs, a time of 180 min is needed instead of seconds to obtain a polyacrylamide hydrogel. After these surprising results obtained for AAm, various acrylic monomers were tested, such as 2-(hydroxy ethyl) acrylate (HEA), 2-(hydroxy ethyl) methacrylate (HEMA), and the ionic monomer 2-[(methacryloyloxy) ethyl] trimethylammonium chloride (METAC). In all cases, fast photopolymerization reactions were observed using the MWCNT/Rf/TEA PIS, leading to homogeneous hydrogels formation in less than 3 min.

**Scheme 1:**
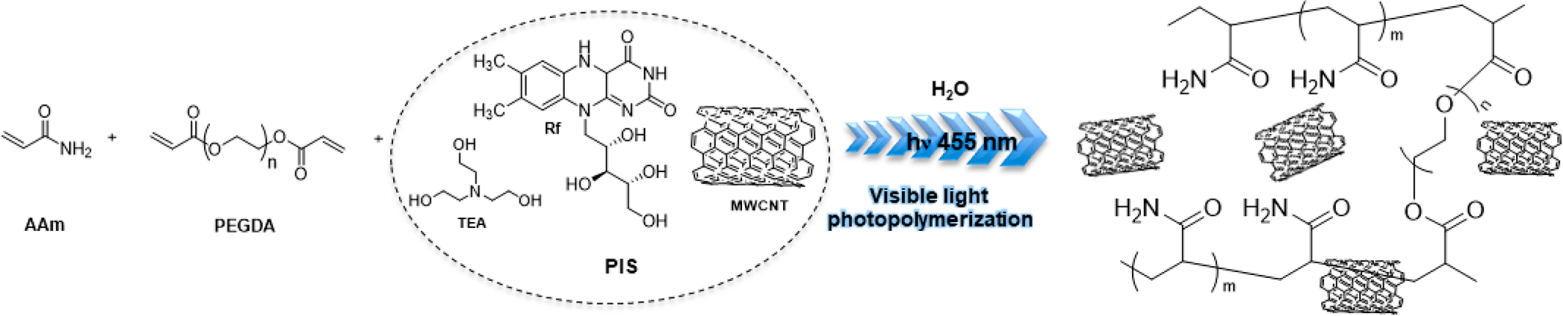
Schematic Representation of the Main Reagents Involved in the Photopolymerization Reaction

The kinetics and the extent of polymerization were followed by near-infrarred (NIR) FTIR, employing the same liquid chamber and homemade photoreactor named before (see SI for more information).^29^ Different prepolymeric mixtures were investigated (Table S1), employing AAm and PEGDA as the monomer and cross-linker, varying the presence and replacement of Rf, TEA, oxygen, and carbon additives (0.25 wt % with respect to AAm) for the evaluation of the proposed new PIS. The prepolymeric mixtures were irradiated with blue LEDs of 455 nm, employing a cutoff UV filter at 400 nm to avoid side reactions promoted by UV light. Figure 1 shows the conversion of vinylic monomers/cross-linkers vs irradiation time followed by analyzing the disappearance of the band at 6182 cm^–1^ associated with the acrylic double bond. Figure S1 shows the FTIR spectrum obtained at different irradiation times, in which the process was stopped every minute to make a measurement. As observed, when the exposure time to light increased, the vinylic peak decreased until almost zero absorption, indicating full conversion around 10 min of irradiation mostly due to the data acquisition time without light. Each point of Figure 1 was obtained by integrating the band at each exposure time from Figure S1 and plotting the C=C absorption band versus irradiation time.

**Figure 1:**
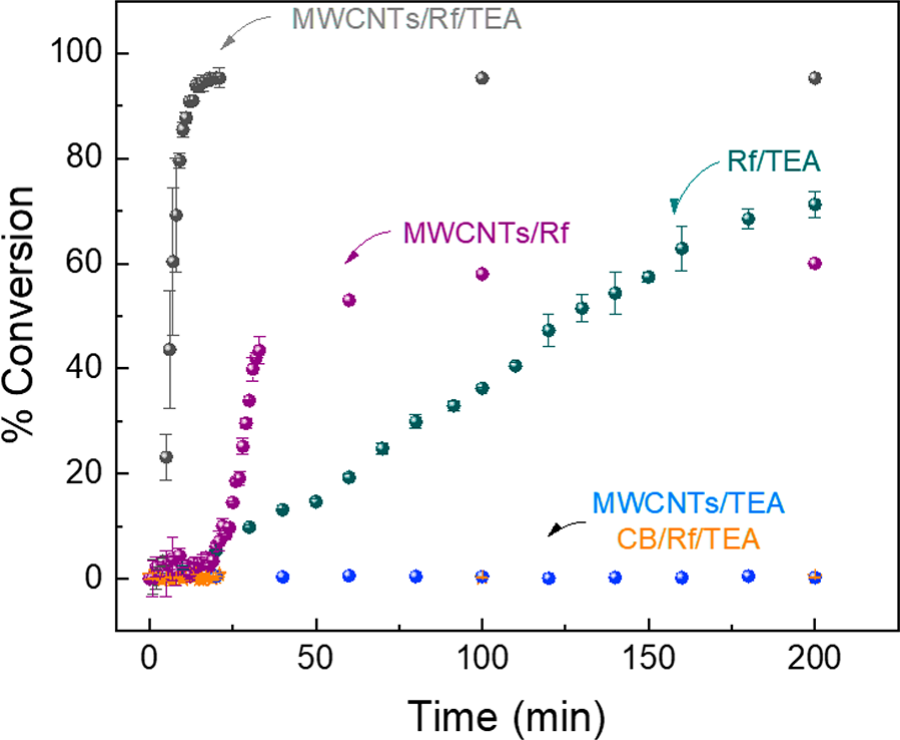
Photopolymerization kinetics given by % acrylamide monomer conversion vs irradiation time (min) of different photoinitiator systems (PISs).

In Figure 1, we compared the kinetic results using different PISs when photopolymerization of the acrylamide monomer was carried out in a liquid chamber of 1 cm optical path. A burst effect was observed for monomer conversion using the initiating system MWCNTs/Rf/TEA in which a total conversion is reached after solidification took place in less than 200 s. The absence of a significant induction period (polymerization started after 4 min) represents an important piece of evidence of the efficiency of the system. Moreover, a monomer conversion up to 70–80% is reached in just 10 min for the MWCNTs/Rf/TEA system, and 100% of conversion is observed in less than 20 min of irradiation. Furthermore, this fast kinetics is observed in the presence of oxygen which is not the typical case for type II photoinitiators where the presence of oxygen normally inhibits the polymerization. As benchmark comparisons and to try to understand the photopolymerization reaction, we carried out several tests. First, we investigated the PIS without MWCNTs employing the same setup. The reference system (Rf/TEA) displays a much lower polymerization rate. Even without oxygen in the media, only a maximum conversion of 70% was reached after 180 min. Second, we investigated the initiating system MWCNT/Rf. In this case, the photopolymerization occurred with higher rate than the reference system; however, only 25% conversion was observed after 20 min, and an extended induction period was observed. We also carried out the photopolymerizations in the presence of other carbon additives such as carbon black (CB) or graphene oxide as substitutes of MWCNTs. In those cases, photopolymerization did not occur at all.^30^ On the other hand, we checked the polymerization in the absence of Rf as a sensitizer, and kinetics experiments were carried out using the MWCNT/TEA system. The polymerization did not occur at all. Finally, we carried out the kinetics experiments, replacing Rf by two commonly used commercial photoinitiators (BAPO and Irgacure 2959) and a dye (Safranine) that can interact by π-stacking with the MWCNTs, all of them to verify the role of Rf and MWCNTs in the PIS. All the molecules that replaced Rf were used in the same concentration (1 × 10^–5^ M), and also BAPO and Irgacure 2959 were tested at 5 wt % of AAm (a commonly used concentration of these photoinitiators). All the kinetic experiments showed that the polymerization did not occur, as observed in Figure S2, showing that the polymerization acceleration only takes place when the new MWCNT/Rf/TEA PIS is employed.

Many factors must be contributing to the important increase in the polymerization rate using the MWCNT/Rf/TEA PIS. On one hand, as known for type II PISs, the photoinitiator system Rf/TEA generates an active amino radical (R^•^) by an electron transfer reaction between the amine and the triplet excited state of the dye (^3^Rf*).^12,31,32^ The amine transfers an electron to ^3^Rf*, and after a fast proton abstraction, amino radicals start the chain reaction of polymerization (Scheme S1a). On the other hand, MWCNTs were employed as radical initiators for photopolymerization as mentioned above, and the mechanism proposed was similar to other semiconductors, where electrons and holes are generated in the conduction and valence band, respectively.^11,18,33,34^ It is reasonable to consider that holes on the surface of MWCNTs could also react with TEA, generating active amino radicals, as proposed in Scheme S1b. However, this fact by itself cannot explain the synergistic effect in the presence of the Rf/TEA visible-light system. Our hypothesis to explain this is to consider also the effect of surface interaction between the MWCNTs and the Rf dye. As is well known, MWCNTs tend to adsorb dyes and electrophilic compounds by π stacking over their surfaces.^35,36^ The organic molecules (Rf and TEA) that act as a typical type II PIS in solution may increase the rate of polymerization due the closeness in the surface of MWCNTs. The UV–vis spectrum through diffuse reflectance was recorded by employing a MWCNT/Rf solution in water to corroborate this hypothesis. The vitamin B2 presents typical absorbance peaks around 374 and 455 nm after the incorporation of MWCNTs in the dye aqueous solution, as shown in Figure S3. After 30 min of MWCNT/Rf solution preparation, the peaks of the sensitizer remain present, and the peaks of the CNT are sharp and intense. This indicates that the bundles of the CNTs are well dispersed, and the CNTs are more individualized in the aqueous solution due to the interaction with vitamin B2. This closeness would avoid diffusional steps, that is, in the electron transfer reaction between the dye and amine to originate the reactive amino radical which is also indicated by the low inhibition time in the presence of oxygen. Finally, the semiconducting MWCNT may also contribute to the generation of radicals and participate in the initiating system (Scheme S1b) where both holes and electrons could react directly with monomers to generate active radicals and produce the chain reaction.^18^

To clarify the role of MWCNTs in the polymerization mechanism, electron paramagnetic resonance (EPR) experiments were carried out. The spectra of aqueous solutions containing the monomers and the different components of the photopolymerizing system with and without MWCNTs were recorded at room temperature, before and after irradiating the samples (see SI). Before irradiation all samples without carbon nanotubes were EPR silent, but after irradiation a weak signal was observed in solutions containing the monomer, the sensitizer (Rf), and the co-initiator (TEA) (Figure S4a). This signal appears to correspond to an amino radical with *g* = 2.0042 and *A*_N_ = 7.8 G. It is well-known that under certain light aqueous solutions of Rf generate very short-lived radicals that can only be detected by using spin traps (DMPO or PBN).^37^ Therefore, the observed signals must correspond to the Rf/TEA system formed prior to the generation of the acrylamide radical that will initiate the chain propagation process. When the equivalent system containing MWCNTs was irradiated, this radical was not observed, confirming the direct participation of the carbon nanotubes in the polymerization mechanism. The associated radical to the MWCNT/Rf/TEA system was not detected because is more rapidly oxidized by the monomers, with the consequent increase of the polymerization rate (Figure S4b). These experiments allowed us to confirm the proposed mechanism in Scheme S1b, where MWCNTs generate active radicals and produce the chain reaction.

In view of the interesting results obtained for the new PIS MWCNT/Rf/TEA, some examples of application were tested, including the synthesis of various CNT nanocomposite hydrogels and 3D printing. For the synthesis of hydrogels, different compositions including varying amounts of pristine MWCNTs (1, 3, and 5 wt %) and AAm/PEGDA were employed to obtain polyacrylamide/CNT nanocomposite hydrogels. Table 1 shows the characterization of the hydrogels in terms of water swelling behavior, mechanical properties, and electronic conductivity.

**Table 1:** Characterization Data of the Polyarylamide/MWCNT Hydrogels

hydrogel	Sw max (%)	*E′* modulus (Pa)	conductivity (mS cm^–1^)
PAAm	1950	6.5 × 10^5^	0.016
PAAm 1 wt % MWCNTs	916	1.3 × 10^6^	0.2
PAAm 3 wt % MWCNTs	790	2.9 × 10^6^	3.3
PAAm 5 wt % MWCNTs	723	1.1 × 10^7^	1.75

Figure S5 presents the % swelling behavior in water vs time. As observed, the incorporation of MWCNTs sharply decreases the water uptake capability of the hydrogels (see Table 1). Even 1 wt % of the CNTs included in the PIS formulation decreased the swelling degree between two and three times with respect to the hydrogels synthesized employing the Rf/TEA system (2000%). The incorporation of 1, 3, or 5 wt % of MWCNTs did not show a big difference, where the swelling ranged from 900 to 700%, being the lowest swelling of the hydrogel with the highest quantity of CNTs. These results are expected due to the presence of a hydrophobic additive that can affect the swelling of the hydrogels. The internal structure of PAAm 1 wt % MWCNTs and PAAm 5 wt % MWCNTs can be observed in Figure S6 through SEM images. For this measurement, the hydrogels were swelled during 24 h before a freeze-drying process in order to observe the differences regarding the internal structure between the different hydrogels. The higher the amount of MWCNTs, the brighter the structure since the presence of MWCNTs provides electrical conductivity reflected in the brightness of the SEM images.

Interestingly, the electronic conductivity of the PAAm/MWCNT nanocomposites was measured through a four-point probe, and its values are summarized in Table 1. As it can be observed, the electric conductivity increases with the amount of MWCNTs in the formulations reaching a value of 175 mS cm^–1^ when 5 wt % of MWCNTs are employed in the formulations. These high electronic conductivity values are comparable to others reported before for conducting hydrogels based on well-dispersed CNTs.^25,38−42^

The presence of MWCNTs also affected the mechanical properties of the PAAm hydrogels, as observed in Figure 2a. The *É* modulus increases from 6.5 × 10^5^ Pa to 1.1 × 10^7^ Pa (at 1 Hz frequency and 25 °C), obtaining a harder hydrogel with the increased amount of MWCNTs. These differences can be observed in Table S1 for all the formulations. Even though the incorporation of CNTs increases the modulus, the final obtained materials are flexible and stretchable, as shown in Figure 2b, which shows pictures of PAAm 1 wt % MWCNT hydrogel handling.

**Figure 2:**
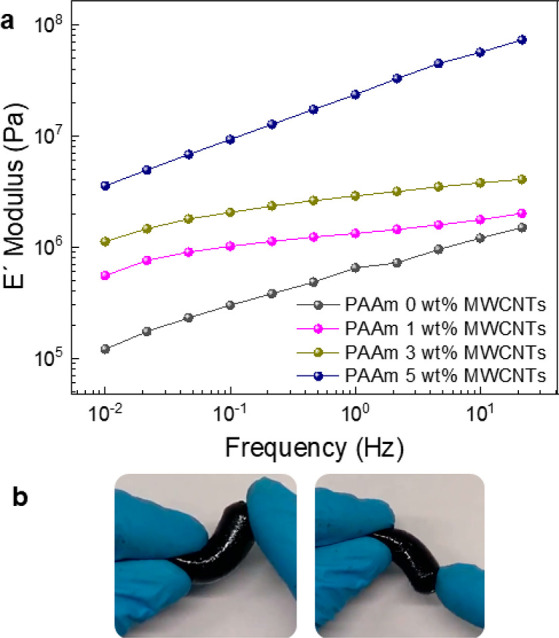
Hydrogel characterization by dynamic mechanical analysis (DMA) (a) of the different hydrogels containing 0, 1, 3, and 5 wt % of MWCNTs. (b) Pictures showing the flexibility of PAAm 1 wt % MWCNT hydrogels.

Finally, the new PIS based on MWCNTs/Rf/TEA was tested for 3D printing in a commercially available LCD 3D printer. Liquid crystal display (LCD)^40^ 3D printing needs a fast and highly efficient PIS to initiate the polymerization without needing a deoxygenated prepolymeric solution. Thus, a formulation including MWCNTs/Rf/TEA with AAm (see Table 1, first line) was sonicated for 15 min and printed, employing a ELEGOO MARS PRO 2 printer (*h*ν 405 nm, UV filter of 385 nm cutoff). Figure 3a shows a schematic representation of the equipment used for the photopolymerization process employing 3D printing, where a platform moves in the *Z* axis, dipping it into the prepolymeric solution. While the laser polymerizes layer by layer, a light source irradiates from bottom up. The height of each print layer was 0.1 mm; the elevation speed employed was 100 mm min^–1^; and the exposure time was 30 s for each layer (for more information, see the SI). It is worth noting that a simple prepolymeric solution composed of conventional acrylic monomers and water is used in our case, compared to the complex commercial formulations including prepolymers and/or oligomers.

**Figure 3:**
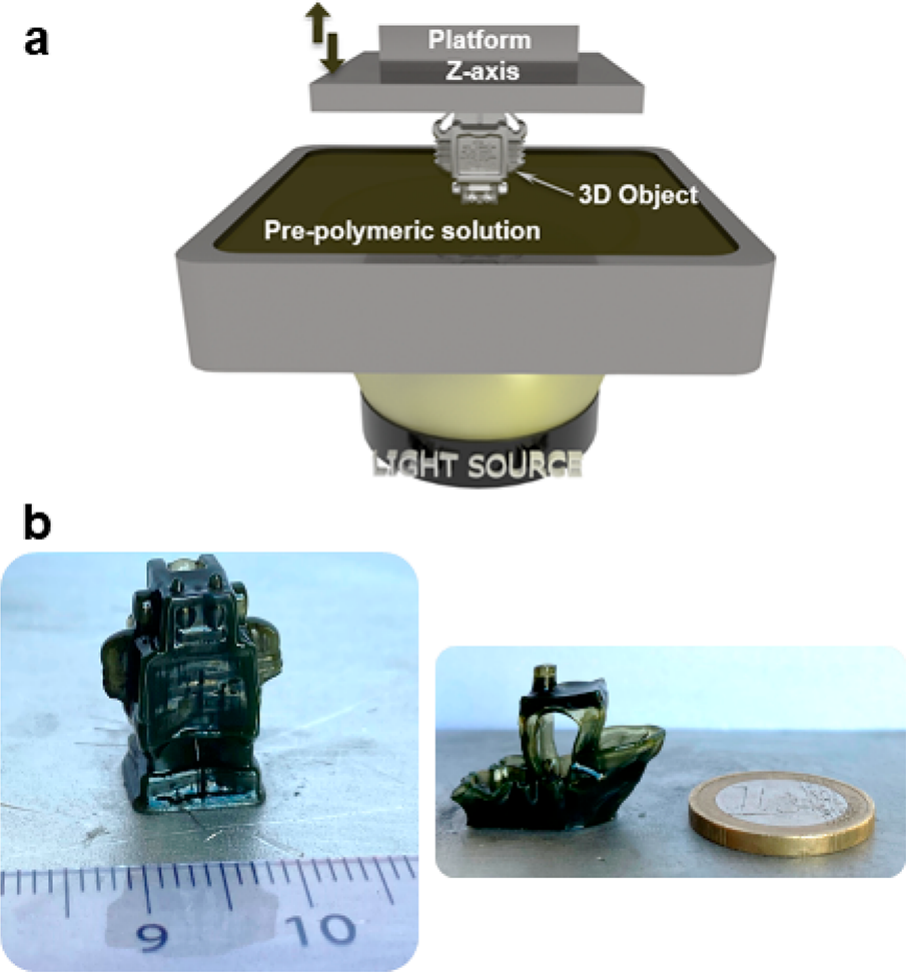
Representative scheme of the 3D printer performance (a) and pictures of some printed 3D objects (b).

Figure 3b shows the obtained printed objects, based on conductive PAAm/MWCNT nanocomposite hydrogels with the shape of a robot (2 cm high; 1 cm wide) and a boat that took between 50 and 60 min to manufacture. The employment of conventional monomers intead of commercial resins, the excellent resolution of the observed details, and the small size of the 3D objects show the great ability of the new proposed PISs to be employed through this printing technique. It is worth noting that CNT-based 3D-printed conducting objects are highly desirable for different applications ranging from tissue engineering to new (bio)electronic devices such as electroactuators, scaffolds for tissue engineering, or sensors.^39,43−48^

In summary, a new photoinitiator system based on MWCNT/Rf/TEA is presented for fast visible-light-induced photopolymerization of acrylic monomers in the presence of carbon nanotubes. Our results indicate that the MWCNTs participate in the photoinitiation process, accelerating the polymerization of acrylamide and other acrylic monomers. This PIS allows us to avoid problems commonly present in this class of photopolymerizations, such as slow polymerizations, the presence of oxygen, or reaching quantitative conversions. As a result, conducting polyacrylamide/MWCNT hydrogel nanocomposites could be obtained quickly, showing excellent mechanical, thermal, and electrical properties. The new PIS was also tested for 3D printing in a commercially available LCD 3D printer, demonstrating its versatility for the manufacture of different 3D-printed objects based on the conductive polymer CNT nanocomposites.

## Supporting Information Available

Materials, synthesis of the prepolymeric solutions, and synthesis of PAAm/MWCNTs hydrogels are described. Swelling characterization, kinetic measurements of photopolymerization, FTIR, UV–vis spectroscopy, EPR, DMA, SEM, four-point probe, and 3D printing methods are explained. Different figures are also incorporated: real-time NIR-FTIR absorbance, UV–vis absorbance of a MWCNT/Rf aqueous solution, proposed mechanism of photopolymerization, swelling behavior of PAAm/MWCNT hydrogels, and SEM images of PAAm/MWCNT hydrogels are also included (PDF)
