# Supplementary material for: Fast Visible-Light Photopolymerization in the Presence of Multiwalled Carbon Nanotubes: Toward 3D Printing Conducting Nanocomposites
Source: ACS Macro Lett. 2022 Feb 10;11(3):303–9. doi: 10.1021/acsmacrolett.1c00758 (PMC8928478; doi:10.1021/acsmacrolett.1c00758)
Supplement: Supplementary file 1 — mz1c00758_si_001.pdf [file mz1c00758_si_001.pdf]

Supporting information

Fast visible-light photopolymerization in the presence of MWCNTs: Towards 3D printing conducting nanocomposites

Antonela Gallastegui[a], Antonio Dominguez-Alfaro[a,b], Luis Lezama[c], Nuria Allegret[b], Maurizio Prato[b,d,f], María L. Gómez[e], David Mecerreyes[a,f].

[a] POLYMAT, University of the Basque Country UPV/EHU, Avenida Tolosa 72, Donostia-San Sebastian, 20018, Gipuzkoa, Spain

[b] Center for Cooperative Research in Biomaterials (CIC biomaGUNE), Basque Research and Technology Alliance (BRTA), 20014 Donostia-San Sebastián, Spain

[c] Departamento de Química Inorgánica, Fac. Ciencias, UPV/EHU, Aptdo. 644, Bilbao, Spain

[d] Department of Chemical and Pharmaceutical Sciences, INSTM unit of Trieste, University of Trieste, Via L. Giorgieri 1, 34127 Trieste, Italy

[e] Instituto de Investigaciones en Tecnologías Energéticas y Materiales Avanzados (IITEMA), Universidad Nacional de Río Cuarto and Consejo Nacional de Investigaciones Científicas y Tecnológicas (CONICET), Campus Universitario, 5800, Río Cuarto, Argentina

[f] IKERBASQUE, Basque Foundation for Science, Bilbao, Spain

Corresponding author: David Mecerreyes (E-mail: david.mecerreyes@ehu.es)

Experimental

Materials

The monomers acrylamide (AAm) (98%), 2-(hydroxy ethyl) acrylate (HEA) (99%), 2-(hydroxy ethyl) methacrylate (HEMA) (99%) and 2-[(methacryloyloxy) ethyl] trimethylammonium chloride (METAC) (80% wt in water) (97%) were obtained from Sigma Aldrich. Vitamine B2 Riboflavine (Rf), triethanolamine (TEA) (99%) and poly(ethylene glycol) diacrylate (PEGDA MW 575) (99%), BAPO (Phenylbis(2,4,6-trimethylbenzoyl)phosphine oxide) and Irgacure 2959 were bought also from Sigma Aldrich.

Multi walled carbon nanotubes (MWCNTs) (95%) were obtained from Nanoamor Inc. (Stock#1237YJS: inner diameter 5−10 nm; outside diameter 20−30 nm; length 0.5−2 µm).

Carbon black C65 (CB) was bought from Timical.

Water was Milli-Q quality.

Reagents were employed with no furder purification except for TEA that was purified by vacuum distillation prior to use.

Methods

Kinetic measurement of photopolymerization

Consumption of C=C groups during photopolymerization was monitored by following the disappearance of the 6182 cm^{-1} absorption peak, as described previously by Stransbury and Dickens. NIR-FTIR absorption spectra were acquired using a NICOLET IS20 XT-KBR DTGS of middle-near infrarred Thermo Scientific spectrophotometer. Pre-polymeric mixture samples were placed in a modified homemade liquid sample chamber. The chamber consists of a silicone spacer (yielding an optical path length = 1 mm) sandwiched between two glass windows pressed by a metal frame using four tightening screws. In situ excitation was performed using a home-made portable irradiator equipped with three blue LEDs (emission maximum = 455 nm, FWHM ~ 50 nm). The irradiator was placed inside the IR spectrometer for sample illumination and was removed immediately before spectra acquisition, every 1 min for the first 20 min. The pre-polymeric mixtures consisted of aqueous solutions having a total of AAm/PEGDA concentration of 50 wt% and crosslinker concentration of 2 wt% respect AAm. The co-initiator TEA was used at a concentration of $1x10^{-3}$ M and Rf was used at a concentration of $1x10^{-5}$ M (or resulting in an absorbance of 0.3 at 455 nm using a 1 cm optical path length cuvette).

Once the carbon additive (0.25 wt% respect AAm concentration) was incorporated into, the pre-polymeric mixtures were sonicated during 15 min prior to the kinetic measurements.

Some extra experiments were also carried out replacing Rf by some photoinitiators normally employed, like BAPO, Irgacure 2959 and Safranine. For all of them, the employed concentration was the same as Rf ($1x10^{-5}$ M) and for BAPO and Irgacure 2959 a 5 wt% respect AAm were also tested.

Hydrogels synthesis

PAAm hydrogels were synthesized employing the same composition as the kinetic measurements. 3 mL of each sonicated pre-polymeric solutions with 0, 1, 3 or 5 wt% of MWCNTs on its composition were placed in cilindrical test tubes of 1 cm of diameter and irradiated during 180 secs at 25 ºC in a homemade merry-go-round photochemical reactor supplied with nine blue LEDs (emission maximum = 455 nm, FWHM ~ 50 nm). A cutoff filter at 400 nm was

employed to avoid the interaction of UV light. After photoirradiation, hydrogels were recovered by breaking the test tubes.

Swelling test

For the swelling measurements, disks (10.5 mm diameter, 2.5 mm thickness) of dried PAAm hydrogels containing 0, 1, 3 or 5 wt% MWCNTs incorporated with the PIS were weighted (W_0) and immersed in Milli Q water during a *t* time. Later, the swelled hydrogels were weighted (W_t) and returned to the water. This process was repeated until a constant weigh was reached.
The swelling percentage was informed as follows:

$$\%Swelling = (\frac{W_0 - W_t}{W_0})\ x\ 100 \qquad (1)$$

Each point of the swelling curve belongs to the average of three individual determinations.

Fourier transform infrared (FTIR)

FTIR absorption spectra of all pre-polymeric solutions for the kinetic measurements during photopolymerization were acquired using a NICOLET IS20 XT-KBR DTGS of middle-near infrared Thermo Scientific spectrophotometer.

UV-Visible spectroscopy

UV-Vis spectroscopy measurements of the MWCNTs/Rf solutions were performed on a Hewlett Packard (HP) model 8453 spectrophotometer.
MWCNTs/Rf solution was prepared in water, Rf was used at a concentration of $1x10^{-5}$ M and 0.25 wt/v% of MWCNTs were added. Once the solution was sonicated during 15 min, we waited 30 min to take the spectrum of the prepeared solution.

Electron Paramagnetic Resonance (EPR)

EPR measurements were performed at room temperature using a Bruker ELEXSYS E500 spectrometer operating at the X-band. The spectrometer was equipped with a super-high-Q resonator ER-4123-SHQ. The magnetic field was calibrated by a NMR probe and the frequency inside the cavity (~9.36 GHz) was determined with an integrated MW-frequency counter. Samples were placed into quartz glass capillary tubes with 0.8 mm inner diameter. Typical instrument settings were: centre field, 3340 G; scan range, 100 G; receiver gain, 30 dB; time constant, 81.92 ms; modulation amplitude, 4.0 G; microwave power, 20 mW, accumulated scans, 20. Data was collected and processed using the Bruker Xepr suite.

Two prepolymeric solutions were prepeared for the analysis: MWCNTs/Rf/TEA and Rf/TEA solutions were prepared in water, Rf was used at a concentration of $1x10^{-5}$ M and 0.25 wt/v% of MWCNTs, when needed, were added. Once the solution was sonicated during 15 min, EPR spectra were taken after irradiation.

Dynamic Mechanical Analysis (DMA)

Dynamic Mechanical Analysis (DMA) was performed using a TA Instruments DMA Q800 apparatus. Scans were conducted from 0.01 to 35 Hz at 25 ºC. Photopolymerized PAAm hydrogels containing 0, 1, 3 or 5 wt% of MWCNTs were cut in a rectangular shape of 2 cm x 1 cm for the measurement.

Scanning Electron Microscopy (SEM)

SEM measurements were performed on a JEOL JSM-6490LV at 5 kV, running in a point-by-point scanning mode. The PAAm hydrogels were swelled during 24 h in MilliQ water at 25 ºC and then freeze dried, in this way the internal structure of the materials could be seen through the technique. The samples were placed on an aluminum holder with double-side carbon tape and introduced in the SEM chamber. All the samples were evaluated at different magnifications.

4-Points probe: Electronic conductivity test

The electronic conductivity measurements of PAAm hydrogels containing different quantities of MWCNTs were performed on a four-point probe Ossila Sheet testing three different sample zones in triplicate. Thin rectangular pieces of approximately 1 mm thickness and 10 mm diameter were tested, and electronic conductivity was calculated, taking into account the thickness of each sample.

3D Printing (Elegoo MARS Pro 2)

The 3D printed objects in this work were designed using the commercial 3D modeling program Chitubox (Chitubox V1.6.1). The .ctb file was sectioned and the data were transmitted to the 3D printer, resulting in a solid product of the 3D model. In the case of Figure 3, the 3D printer employed was an Elegoo MARS Pro 2 and the 3D procedure employed was throught Digital light Processing (DLP).

Table S1. Composition of pre-polymeric mixtures test by photopolymerization.

Pre-polymeric mixtures	Rf	TEA	AAm/PEGDA	Desoxygenation	Carbon additive
MWCNTs/Rf/TEA	$1x10^{-5}$ M	$1x10^{-3}$ M	50 wt%	-	0.25 wt% MWCNTs
CB/Rf/TEA	$1x10^{-5}$ M	$1x10^{-3}$ M	50 wt%	-	0.25 wt% CB
Rf/TEA	$1x10^{-5}$ M	$1x10^{-3}$ M	50 wt%	15 min	-
Rf	$1x10^{-5}$ M	-	50 wt%	15 min	-
BAPO	$1x10^{-5}$ M / 5 wt%	-	50 wt%	-	0.25 wt% MWCNTs
Irgacure 2959	$1x10^{-5}$ M / 5 wt%	-	50 wt%	-	0.25 wt% MWCNTs
Safranine	$1x10^{-5}$ M	-	50 wt%	-	0.25 wt% MWCNTs

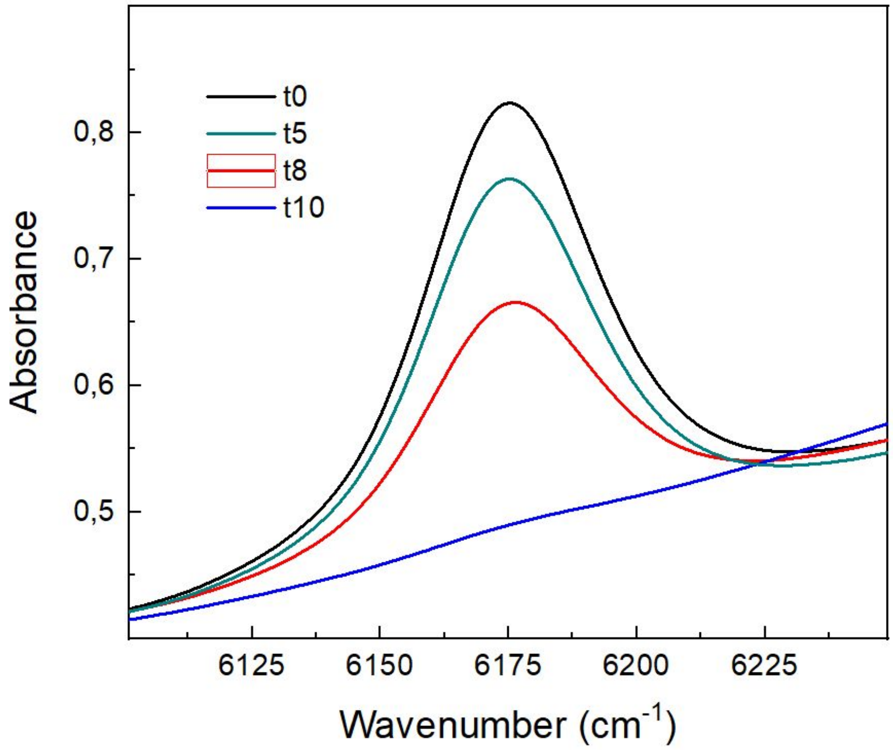

Figure S1. FTIR absorbance band center at 6182 cm^{-1} regarding the vinylic peak of AAm after 0, 5, 8 and 10 min of irradiation.

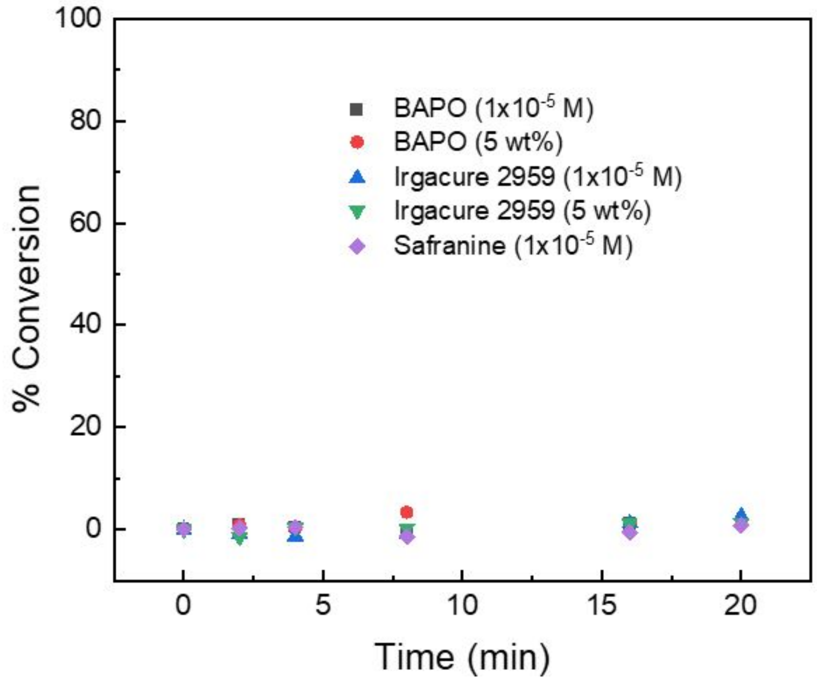

Figure S2. Photopolymerization kinetics given by % acrylamide monomer conversion vs irradiation time (min) in pre-polymeric solutions where Rf was repleaced by photoinitiators commonly used employing two different concentrations (BAPO and Irgacure 2959) and Safranine, a molecule that may interact by π-stacking with MWCNTs.

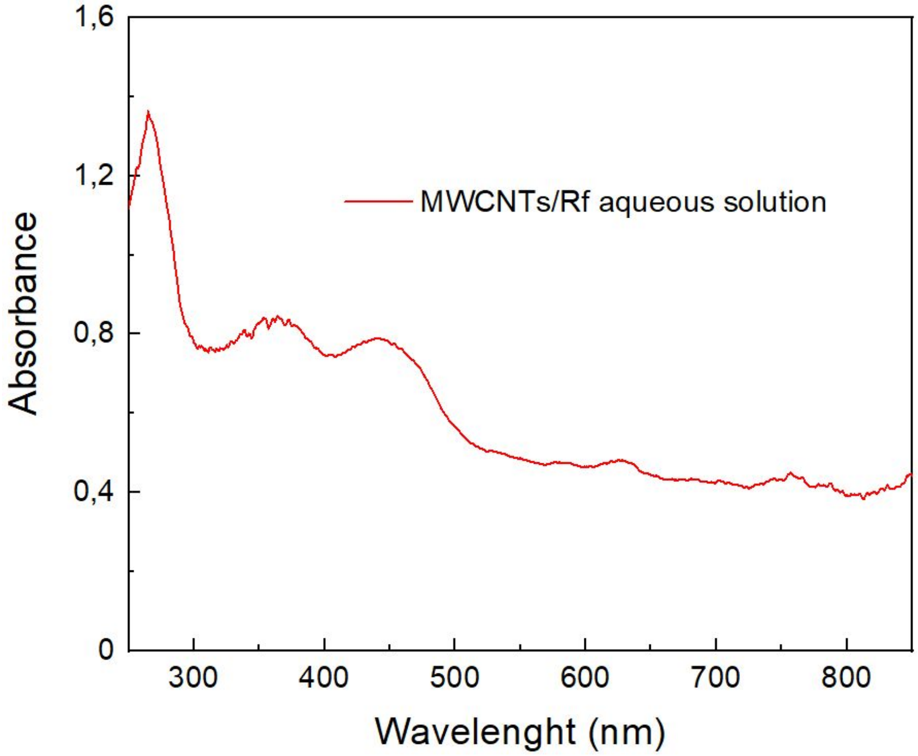

Figure S3. UV-Vis spectrum of a MWCNTs/Rf aqueous solution after 30 min of its preparation.

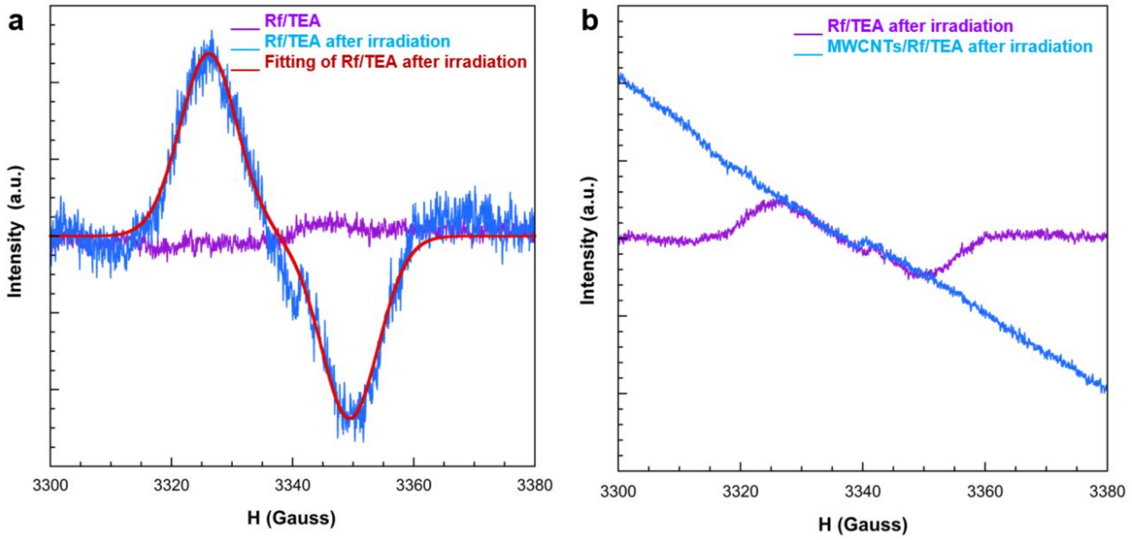

Figure S4. EPR experiments for the Rf/TEA PIS before and after irradiation (fitting after irradiation employing the data g=2.0042 and A_N=7.8 G from amino radicals) (a); EPR experiments comparing Rf/TEA and MWCNTs/Rf/TEA PIS after irradiation.

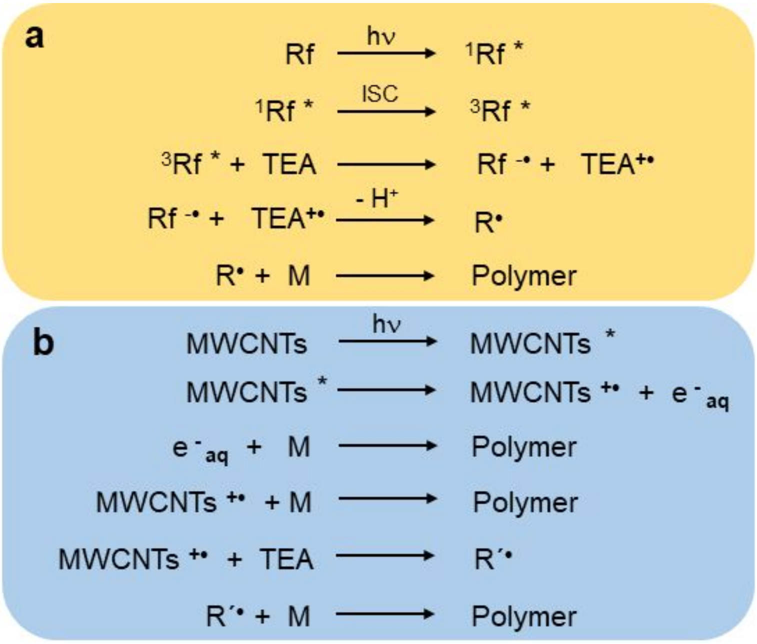

Scheme S1. Visible light photoinitiating mechanism for vinyl polymerization employing the known photoinitiator system Rf/TEA Type II (a) and the proposed paths when MWCNTs intervene in the process.

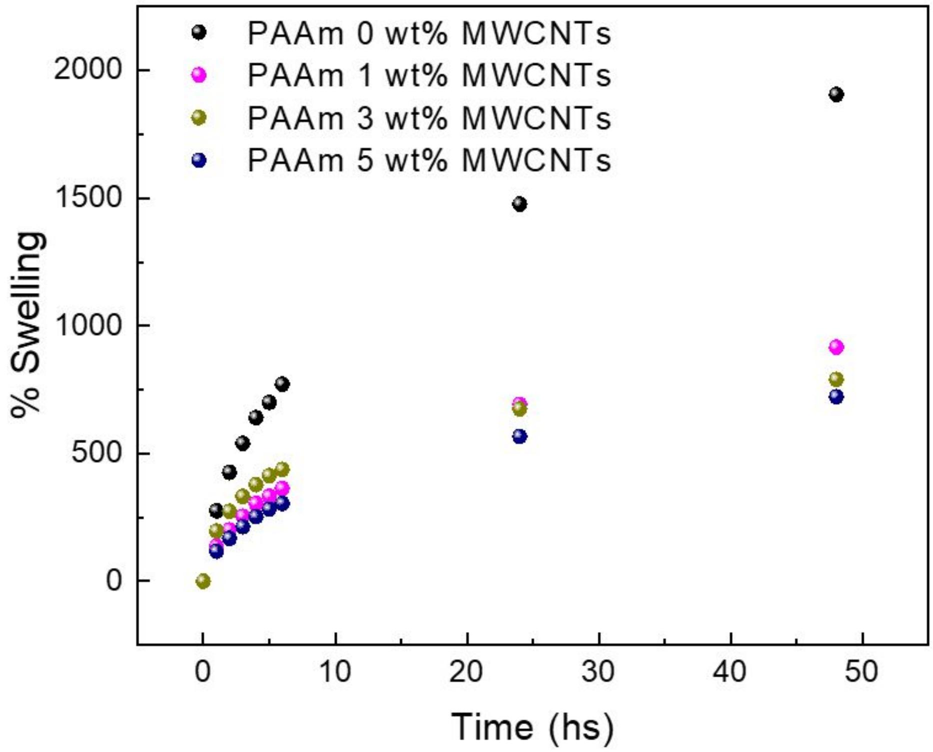

Figure S5. Swelling (%) in water vs time of PAAm hydrogels containing 0, 1, 3 and 5 wt% of MWCNTs.

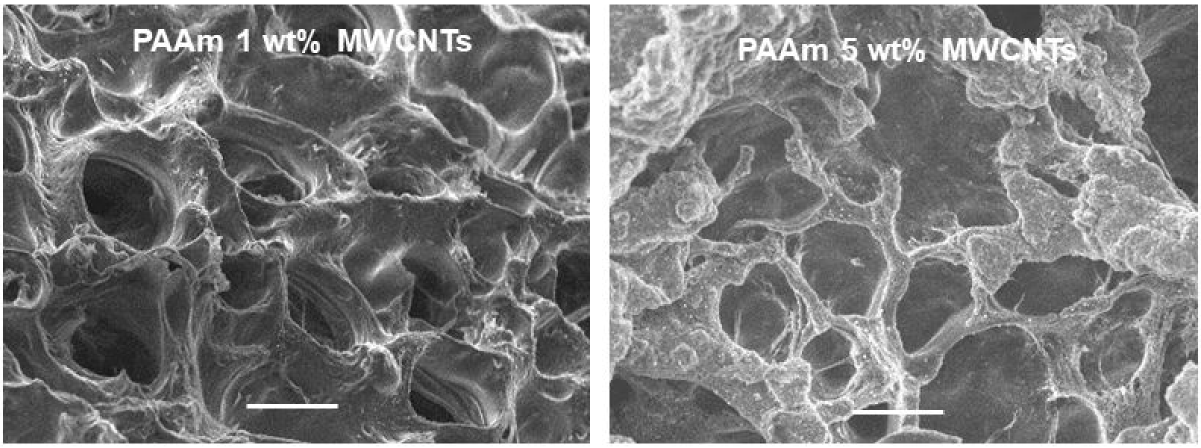

Figure S6. Images of the internal structure given by SEM (100 μm length bar) of the PAAM hydrogels containing 1 (left) and 5 wt% (right) of MWCNTs in the PIS.
